# Complications Related to Urgent Initiation of Peritoneal Dialysis in a Mexican Hospital with Limited Resources: A Prospective Cohort

**DOI:** 10.3390/clinpract16040073

**Published:** 2026-04-13

**Authors:** Camila Baas-Yama, Eduardo Rivera-Huerta, Ivan Zepeda-Quiroz, Carlos A. Guzmán-Martín, Demian Trueba-Lozano, Sebastian Toledo-Ramirez, Ana Ortega-Gonzalez, Irma Archundia-Riveros, Brenda Barrera-Mota, María Jimenez-Baez, Ciria Vázquez-Macias, Daniel Juárez-Villa

**Affiliations:** 1 Department of Internal Medicine and Nephrology, Hospital General de Zona 18, Instituto Mexicano del Seguro Social, Playa del Carmen 77725, Quintana Roo, Mexico; camilabaas3@gmail.com (C.B.-Y.); riveralalo8@gmail.com (E.R.-H.); gabsortega2105@gmail.com (A.O.-G.); cgvazquezm@gmail.com (C.V.-M.); 2 Trauma Surgery Department, Hospital de Traumatología y Ortopedia Dr. y Gral. Rafael Moreno Valle, Public Health Services Instituto Mexicano del Seguro Social para el Bienestar, Puebla 72573, Puebla, Mexico; ivanquiroz621@gmail.com (I.Z.-Q.); dr.trueba87@gmail.com (D.T.-L.); 3 Department of Nephrology, Hospital Ángeles Puebla, Puebla 72190, Puebla, Mexico; 4 Research Programs Department, Shriners Hospitals México, Mexico City 04600, Ciudad de México, Mexico; gmcarlos93@gmail.com; 5 Department of Internal Medicine, Hospital General Regional 17, Instituto Mexicano del Seguro Social, Cancun 77533, Quintana Roo, Mexico; sebastian.toledo262@gmail.com (S.T.-R.); dra.archundia@gmail.com (I.A.-R.); 6 Planning and Institutional Liaison Coordinator, Hospital General Regional 17, Instituto Mexicano del Seguro Social, Playa del Carmen 77533, Quintana Roo, Mexico; brenda.barrera@imss.gob.mx (B.B.-M.); valeria.jimenezb@imss.gob.mx (M.J.-B.); 7 Department of Nephrology, Amerimed Playa del Carmen Hospital, Playa del Carmen 77712, Quintana Roo, Mexico

**Keywords:** urgent peritoneal dialysis, complications, chronic kidney disease, leakage, observational study, public hospital

## Abstract

**Background**: Urgent-start peritoneal dialysis (UPD) has emerged as an alternative modality for initiating kidney replacement therapy when immediate hemodialysis is not available. However, early initiation after catheter placement may increase the risk of mechanical complications. Evidence from real-world settings, particularly in resource-limited healthcare systems, remains limited. **Objective**: To determine the frequency of early complications associated with urgent-start peritoneal dialysis and to identify clinical factors associated with their occurrence. **Methods**: We conducted a prospective observational cohort study including adult patients with chronic kidney disease who initiated peritoneal dialysis within 14 days after catheter placement at a public hospital in Mexico. Patients were followed for 30 days after dialysis initiation. The primary outcome was the occurrence of any dialysis-related complication within 30 days after initiation of peritoneal dialysis. Comparisons were performed according to dialysis initiation timing (<72 h vs. ≥72 h). Multivariable logistic regression was used to identify independent predictors of complications. **Results**: Sixty-five patients were included, of whom 29 (44.6%) developed complications within the first 30 days. Mechanical complications predominated, particularly pericatheter leakage (18.5%) and drainage failure (10.8%). Patients who initiated dialysis within 72 h after catheter placement experienced a significantly higher complication rate. In multivariable analysis, initiation of peritoneal dialysis within <72 h remained independently associated with complications (OR 5.75, 95% CI 1.06–31.29, *p* = 0.043). **Conclusions**: Initiating peritoneal dialysis within 72 h after catheter placement was associated with a significantly increased risk of early complications. When clinically feasible, delaying dialysis initiation beyond 72 h may reduce mechanical complications in urgent-start peritoneal dialysis programs.

## 1. Introduction

Chronic kidney disease (CKD) is defined as a persistent alteration in kidney structure or function lasting at least three months, manifested by a reduction in glomerular filtration rate (GFR) and/or evidence of kidney damage, such as albuminuria or structural abnormalities of the kidney [1,2].

In Mexico, CKD constitutes an important public health challenge. National epidemiological data estimate a prevalence of 9184.9 cases per 100,000 inhabitants, with regional variations across the country [3]. CKD is currently among the leading causes of death nationwide, and the burden of disease continues to increase due to the high prevalence of T2D and hypertension [3,4]. The Mexican Institute of Social Security (IMSS), which provides healthcare coverage to a large proportion of the population, manages approximately 80% of patients receiving kidney replacement therapy (KRT) in the country, and more than half of these patients undergo peritoneal dialysis (PD) as their primary dialysis modality [5,6].

CKD represents a major global health burden, affecting approximately 8–16% of the adult population worldwide and accounting for an estimated 844 million cases globally [7]. The prevalence of CKD continues to rise, largely driven by the increasing incidence of type 2 diabetes mellitus (T2D), hypertension, and population aging [7]. Despite its high prevalence, early stages of CKD often remain undiagnosed, and it is estimated that more than 90% of individuals with early-stage disease are unaware of their condition, resulting in delayed treatment and increased risk of complications and mortality [7,8].

Early detection and management of CKD may delay disease progression and reduce the need for KRT; however, many patients are diagnosed at advanced stages when immediate dialysis initiation is required [8,9]. In this context, urgent-start peritoneal dialysis (USPD) has emerged as an important therapeutic strategy for patients who require rapid initiation of dialysis but do not have immediate access to hemodialysis or a mature vascular access [9]. According to the International Society for Peritoneal Dialysis (ISPD), conventional PD is ideally initiated at least two weeks after catheter placement to reduce the risk of mechanical complications [10]. However, in clinical practice many patients require dialysis initiation earlier than this recommended period. Dialysis initiation within 14 days after catheter placement is generally defined as urgent-start PD [11].

Urgent-start PD has become increasingly utilized in many healthcare systems, particularly in resource-limited settings where access to hemodialysis may be restricted [9,10,11]. In some centers, patients may require dialysis initiation within the first 72 h after catheter insertion due to severe metabolic derangements or uremic symptoms. Consequently, urgent-start PD is often categorized into early urgent-start (<72 h after catheter placement) and delayed urgent-start (72 h to 14 days after catheter insertion) [11,12]. Clinical indications for urgent dialysis initiation typically include severe uremic syndrome, refractory volume overload, electrolyte disturbances such as hyperkalemia, or other complications associated with advanced kidney failure [12].

Complications associated with peritoneal dialysis can be broadly classified as mechanical or infectious. Infectious complications include peritonitis, exit-site infection, and tunnel infection, most frequently caused by organisms such as *Staphylococcus aureus* [13,14]. Mechanical complications may include pericatheter leakage, catheter migration, drainage failure, or catheter obstruction, and are influenced by factors such as catheter placement technique, timing of dialysis initiation, dialysate volume, and patient positioning during dialysis exchanges [13,14,15]. Previous studies evaluating urgent-start PD have reported variable complication rates, with mechanical complications generally occurring more frequently during the early period after dialysis initiation [16,17,18,19].

Although urgent-start PD has been increasingly implemented worldwide, most available evidence originates from centers in high-income countries with well-established dialysis programs. Data describing the frequency and characteristics of early complications in urgent-start PD programs in resource-limited healthcare settings remain relatively scarce, particularly in Latin American populations.

Therefore, the primary objective of this study was to determine the most frequent complications associated with urgent initiation of peritoneal dialysis in a public hospital in Mexico. Secondary objectives included evaluating potential risk factors associated with these complications and determining whether these events resulted in death, transfer to hemodialysis, or the need for surgical reintervention.

## 2. Materials and Methods

### 2.1. Study Design and Reporting

This study was designed as a single-center, prospective observational cohort study. The manuscript was prepared in accordance with the Strengthening the Reporting of Observational Studies in Epidemiology (STROBE) guidelines for observational research. A completed STROBE checklist is provided in Supplementary Material S1.

### 2.2. Study Setting and Population

The study was conducted at a secondary-level public hospital of the Mexican Institute of Social Security (Instituto Mexicano del Seguro Social, IMSS) in Playa del Carmen, Quintana Roo, Mexico. Adult patients (≥18 years) with chronic kidney disease requiring urgent initiation of kidney replacement therapy and undergoing peritoneal dialysis catheter placement were consecutively recruited between November 2024 and May 2025. Eligible participants were those who initiated peritoneal dialysis within 14 days after catheter placement, while patients requiring rescue hemodialysis prior to peritoneal dialysis initiation were excluded. During the study period, 71 patients were assessed for eligibility, of whom six were excluded due to prior rescue hemodialysis, resulting in a final cohort of 65 patients (Supplementary Figure S1). Data were collected prospectively, and a study flow diagram is provided in the Supplementary Materials. The study protocol was approved by the Local Health Research Committee No. 2301 (approval code: R-2024-2301-038) and conducted in accordance with the Mexican General Health Law on Health Research and the Declaration of Helsinki. Given the minimal-risk classification, written informed consent was obtained from all participants.

### 2.3. Peritoneal Dialysis Initiation and Clinical Management

The timing of peritoneal dialysis initiation and dialysis prescription were determined by the treating nephrologist based on the patient’s clinical condition and biochemical parameters. For analytical purposes, patients were categorized according to the interval between catheter placement and dialysis initiation: early initiation (<72 h) and delayed initiation (≥72 h). Clinical and dialysis-related variables recorded included catheter placement technique, indication for urgent dialysis initiation, time from catheter insertion to dialysis initiation (hours), initial dialysate volume, number of exchanges, and biochemical laboratory parameters at baseline.

### 2.4. Outcomes and Definitions

The primary outcome was the occurrence of any complication within 30 days after initiation of peritoneal dialysis.

Complications included mechanical and infectious events associated with urgent-start peritoneal dialysis. The following operational definitions were used:

* Pericatheter leakage: Dialysate leakage through the catheter exit site or subcutaneous tunnel requiring a reduction in dialysate volume, temporary interruption of dialysis, or surgical intervention.
* Catheter translocation: Radiologically confirmed displacement of the catheter tip from the pelvic cavity associated with impaired dialysis function.
* Drainage failure: Inadequate dialysate drainage during exchanges requiring catheter repositioning or surgical management.
* Catheter dysfunction: Mechanical malfunction of the catheter interfering with effective dialysis exchanges.
* Peritonitis: Defined according to International Society for Peritoneal Dialysis (ISPD) diagnostic criteria.
* Exit-site infection: Clinical evidence of infection at the catheter exit site requiring antimicrobial treatment.
* Bleeding: Clinically significant hemorrhage associated with catheter insertion.
* Visceral perforation: Inadvertent injury to abdominal viscera during catheter placement confirmed clinically or radiologically.
* Secondary outcomes included whether complications resulted in death, transfer to hemodialysis, or surgical reintervention. Clinical data were obtained from both physical and electronic medical records.

In addition, previous abdominal surgery was defined as any documented prior abdominal procedure, including both major (e.g., laparotomy) and minor interventions (e.g., laparoscopic procedures), as recorded in the medical records.

### 2.5. Bias Assessment

Given the observational nature of the study, potential sources of bias were considered. Selection bias may have occurred because the timing of dialysis initiation was determined by the treating physician according to clinical urgency. Confounding by indication was anticipated, particularly in patients with greater metabolic or uremic severity who required earlier dialysis initiation. To partially account for this, clinically relevant variables including blood urea nitrogen levels were evaluated in multivariable analyses.

### 2.6. Sample Size Considerations

A convenience sample was used, including all eligible patients who met the inclusion criteria during the study period. No formal sample size calculation was performed because the study was designed as an exploratory prospective cohort, and the number of patients undergoing urgent-start peritoneal dialysis at the study center was limited.

### 2.7. Statistical Analysis

Descriptive statistics were used to summarize the characteristics of the study population. Categorical variables were expressed as frequencies and percentages, whereas continuous variables were described using means and standard deviations or medians with interquartile ranges according to data distribution. Normality of continuous variables was evaluated using the Kolmogorov–Smirnov test.

For bivariate comparisons, patients were stratified according to the presence or absence of complications within the first 30 days after initiation of peritoneal dialysis. Categorical variables were compared using the chi-square test or Fisher’s exact test when appropriate. Continuous variables were compared using Student’s t test or the Mann–Whitney U test, depending on the distribution of the data.

To identify predictors of complications, univariate logistic regression analyses were performed for selected clinical and dialysis-related variables. Variables considered clinically relevant or showing potential association in univariate analyses (*p* < 0.20) were included in a multivariable logistic regression model using the enter method. The final model included dialysis initiation timing (<72 h vs. ≥72 h after catheter placement), age, and blood urea nitrogen (BUN) levels.

Results were reported as odds ratios (OR) with 95% confidence intervals (95% CI). Model performance was evaluated using the omnibus test of model coefficients, the Hosmer–Lemeshow goodness-of-fit test, and pseudo-R^2^ statistics (Cox–Snell and Nagelkerke). A two-sided *p* value < 0.05 was considered statistically significant.

All statistical analyses were performed using IBM SPSS Statistics version 25 (IBM Corp., Armonk, NY, USA).

## 3. Results

During the study period, 71 patients were assessed for eligibility. Six patients required rescue hemodialysis prior to peritoneal dialysis initiation and were therefore excluded. The final cohort consisted of 65 patients with chronic kidney disease who underwent urgent-start peritoneal dialysis.

Women represented 50.8% (*n* = 33) of the cohort. The mean age was 54.75 ± 9.62 years, with a mean height of 160 ± 11.2 cm and mean body weight of 72.48 ± 12.20 kg. The most frequent comorbidities were arterial hypertension and type 2 diabetes mellitus, each present in 96.9% (*n* = 63) of patients. Type 2 diabetes mellitus was the most common cause of CKD, accounting for 84.6% (*n* = 55) of cases, followed by obstructive uropathy (6.2%, *n* = 4). The median time between CKD diagnosis and initiation of peritoneal dialysis was 10 months (range 1.5–36). The primary indication for urgent dialysis initiation was uremic syndrome, observed in 64.6% (*n* = 42) of patients, followed by refractory fluid overload (29.2%, *n* = 19). The median interval between catheter placement and the first dialysis exchange was 37 h (IQR 31.5–44.5). The initial dialysate volume was 1000 mL (range 1000–1500), with a median of six exchanges (range 5–8) within 24 h.

Baseline biochemical parameters included a mean serum creatinine of 10.38 ± 4.34 mg/dL, blood urea nitrogen (BUN) 115 ± 47 mg/dL, serum sodium 133 ± 7.5 mmol/L, potassium 5.3 ± 1.28 mmol/L, glucose 125 ± 51 mg/dL, and hemoglobin 8.74 ± 1.70 g/dL. Baseline characteristics of the study population are summarized in Table 1.

### 3.1. Complications

Within the first 30 days after peritoneal dialysis initiation, complications were observed in 29 patients (44.6%).

The most frequent complication was pericatheter leakage, occurring in 18.5% (*n* = 12) of patients. Other complications included drainage failure (10.8%, *n* = 7), catheter translocation (6.2%, *n* = 4), peritonitis (4.6%, *n* = 3), and tunnel infection (4.6%, *n* = 3).

### 3.2. Comparison Between Patients with and Without Complications

Clinical and procedural characteristics were compared between patients who developed complications and those who did not (Table 2).

The only variable significantly associated with complications was the time interval between catheter placement and initiation of peritoneal dialysis. Patients who developed complications initiated dialysis earlier than those without complications, with a median initiation time of 37 h (IQR 33–43) compared with 49 h (IQR 36–78) among patients without complications (*p* = 0.014).

When analyzed categorically, 79.3% of patients who developed complications-initiated dialysis within 48 h, compared with 50% of patients without complications (*p* = 0.02).

Similarly, 93.1% of patients with complications-initiated dialysis within 72 h after catheter placement, compared with 72.2% of patients without complications (*p* = 0.03).

No significant differences were observed between groups in age, body mass index, baseline biochemical parameters, dialysis prescription, or other clinical characteristics.

### 3.3. Association Between Dialysis Timing and Biochemical Parameters

Patients who initiated dialysis within 72 h after catheter placement had significantly higher BUN levels compared with those who initiated dialysis after ≥72 h (109.44 ± 45.72 mg/dL vs. 77.64 ± 31.39 mg/dL; *p* = 0.008), suggesting greater uremic severity among patients requiring earlier dialysis initiation (Supplementary Figure S2).

### 3.4. Multivariable Analysis

Patients who initiated peritoneal dialysis within 72 h after catheter placement experienced a higher frequency of complications compared with those who started dialysis later. To evaluate predictors of complications, a multivariable logistic regression model was constructed, including dialysis initiation timing (<72 h vs. ≥72 h), age, and blood urea nitrogen levels (Table 3).

The overall model was statistically significant (χ^2^ = 9.48, *p* = 0.024), indicating that the included variables improved prediction of complications compared with the null model. Model calibration was acceptable according to the Hosmer–Lemeshow goodness-of-fit test (*p* = 0.319). The model explained approximately 13.6% of the variance in complications according to the Cox–Snell pseudo-*R*^2^ and 18.2% according to the Nagelkerke pseudo-*R*^2^.

After adjustment for age and BUN levels, initiation of peritoneal dialysis within 72 h after catheter placement remained independently associated with a higher likelihood of complications (OR = 5.75, 95% CI 1.06–31.29, *p* = 0.043). Age showed a borderline association with complications (OR = 1.06 per year, 95% CI 1.00–1.12, *p* = 0.051), whereas BUN levels were not significantly associated with the outcome (*p* = 0.805).

The model correctly classified 66.2% of cases overall, with a sensitivity of 62.1% and specificity of 69.4%.

### 3.5. Clinical Outcomes of Patients with Complications

Among the 29 patients who developed complications, conservative management was successful in 14 cases.

Three patients declined further medical or surgical management. Eight patients required transfer to hemodialysis; of these, six remained on permanent hemodialysis, while two were able to return to peritoneal dialysis after temporary hemodialysis support.

Within 30 days after PD initiation, four patients died. Two deaths were attributed to stress-related gastric ulcer perforation, and two were due to septic shock from infections not related to urgent-start peritoneal dialysis.

**Table 3:** Multivariable logistic regression analysis of predictors of complications.

Variable	β (B)	SE	Wald	df	*p*-Value	OR (Exp(B))	95% CI Lower	95% CI Upper
PD initiation < 72 h	1.749	0.865	4.092	1	0.043	5.748	1.056	31.29
Age (years)	0.056	0.029	3.811	1	0.051	1.057	1	1.118
BUN (mg/dL)	0.001	0.006	0.061	1	0.805	1.001	0.99	1.013
Constant	−4.798	1.843	6.778	1	0.009	0.008	—	

Abbreviations: SE, standard error; OR, odds ratio; CI, confidence interval; BUN, blood urea nitrogen; df; degrees of freedom.

## 4. Discussion

In this prospective cohort study evaluating urgent-start peritoneal dialysis (UPD) in a Mexican public hospital, we observed that nearly half of the patients (44.6%) developed complications within the first 30 days after dialysis initiation. Mechanical complications were the most frequent, particularly pericatheter leakage and drainage failure. Importantly, the interval between catheter placement and initiation of peritoneal dialysis emerged as the only variable significantly associated with complications. Patients who initiated dialysis within 72 h after catheter placement experienced a higher frequency of complications compared with those who started later [19,20].

The demographic and clinical characteristics of our cohort are broadly consistent with previously reported Mexican populations undergoing renal replacement therapy. Type 2 diabetes mellitus and systemic arterial hypertension were the predominant comorbidities, present in 96.9% of patients, which aligns with national reports indicating diabetes as the leading cause of chronic kidney disease in Mexico [3,7]. The prevalence of obesity and the mean BMI observed in our cohort were also comparable to those reported in other Mexican studies [17]. However, the proportion of patients with a prior history of abdominal surgery in our sample differed from that reported in other cohorts, where approximately 23% of patients have undergone previous abdominal procedures [15].

The primary indication for urgent dialysis initiation in our population was uremic syndrome, followed by refractory fluid overload and hyperkalemia. These findings are consistent with reports from other countries, although some studies have documented an even higher proportion of patients initiating urgent dialysis due to uremic symptoms [18,19,20]. Patients who initiated dialysis earlier in our cohort had significantly higher blood urea nitrogen levels, suggesting greater metabolic severity and clinical urgency.

The median interval between catheter placement and dialysis initiation in our study was approximately 37 h, which is shorter than that reported in several international studies where dialysis is typically initiated around four days after catheter placement [19,20]. Early initiation of dialysis may increase the risk of mechanical complications due to insufficient tissue healing around the catheter site, particularly when larger dialysate volumes or multiple exchanges are used during the first days of therapy [11,19,21,22,23,24]. In our cohort, the median fill volume was 1000 mL with six exchanges per day, parameters that may also influence the risk of dialysate leakage.

The overall complication rate observed in our study (44.6%) was higher than that reported in many previous studies, where rates between 19% and 29% have been described [16,17,24]. Pericatheter leakage was the most frequent complication in our cohort, occurring in 18.5% of patients. Although leakage rates of 6–15% have been reported in other urgent-start PD studies [11,19,25], differences in dialysis prescription, catheter placement technique, and patient characteristics may explain the higher incidence observed in our population. Conversely, the frequency of drainage failure in our cohort was lower than that reported in some studies.

In our multivariable analysis, early initiation of peritoneal dialysis (<72 h after catheter placement) was the only independent predictor of complications, increasing the odds of early complications nearly sixfold. Similar findings have been reported in other studies evaluating urgent-start PD, where earlier dialysis initiation was associated with higher rates of leakage and catheter migration [26,27,28,29]. However, these results should be interpreted cautiously, as earlier initiation of dialysis may reflect greater clinical severity rather than a direct causal relationship. In our cohort, patients initiating dialysis earlier had significantly higher BUN levels, suggesting that confounding by indication may partially explain the observed association.

Most complications in our study occurred during the first days after dialysis initiation, with mechanical complications predominating. Infectious complications such as peritonitis and tunnel infection were relatively uncommon during the first 30 days. This temporal pattern is consistent with previous studies reporting that early complications after urgent-start PD are primarily mechanical, whereas infectious complications tend to occur later during follow-up [24,27].

Catheter placement technique may also influence complication rates. In our study, all catheters were placed using an open surgical technique, which limited comparisons with other insertion methods. Previous studies have suggested that percutaneous or modified Seldinger techniques may be associated with lower rates of mechanical complications and catheter migration [30,31]. These approaches have also been evaluated in Mexican populations and may represent a promising alternative for reducing early complications in urgent-start PD programs [15].

### Clinical Practice Perspectives

Our findings highlight several practical considerations for urgent-start PD programs, particularly in resource-limited settings. First, whenever clinically feasible, delaying dialysis initiation for more than 72 h after catheter placement may reduce the risk of early mechanical complications. Second, optimization of dialysis prescriptions during the first days of therapy, including lower dialysate volumes and careful patient positioning, may help minimize leakage and catheter dysfunction.

Future technological innovations, including digital monitoring tools and predictive analytics, may further improve patient monitoring and complication prediction in peritoneal dialysis programs [32,33,34,35]. However, the implementation of these technologies remains challenging in many low-resource settings due to limitations in infrastructure, connectivity, and digital literacy among patients and healthcare providers.

The management of patients undergoing peritoneal dialysis also requires a multidisciplinary approach involving nephrologists, surgeons, and specialized nephrology nursing teams. Nursing staff play a key role in early monitoring of complications, patient education, and training of patients and caregivers in dialysis techniques, which are essential components for successful long-term PD therapy. Patient-centered approaches and shared decision-making have also been recognized as important components of renal replacement therapy programs, improving patient engagement and treatment adherence [36]. In addition, patients initiating renal replacement therapy frequently present with complex clinical conditions, including frailty and nutritional alterations. Frailty has been increasingly recognized in patients receiving peritoneal dialysis and may influence treatment outcomes and complication risk [37]. Similarly, nutritional status and targeted medical nutrition therapy play an important role in maintaining functional status and improving clinical outcomes in patients undergoing peritoneal dialysis [38]. These factors should be considered when designing comprehensive care strategies for patients initiating urgent-start PD.

## 5. Limitations

This study has several limitations. First, the relatively small sample size and single-center design may limit the generalizability of the findings. Second, the study used a convenience sample without formal sample size calculation, reflecting the limited number of patients undergoing urgent-start PD at the study center. Third, dialysis prescriptions were not fully standardized, which may have influenced the incidence of mechanical complications such as leakage. Another important limitation is the potential for confounding by indication. Patients who initiated dialysis earlier may have had greater metabolic or clinical severity, as suggested by higher BUN levels, which could independently increase the risk of complications. Although multivariable analysis was performed, the model included only a limited number of variables due to the small number of events. On the other hand, catheter placement was performed exclusively using an open surgical technique, preventing comparisons with other insertion methods that may have different complication profiles. Finally, the unusually high prevalence of prior abdominal surgery (98.5%), which may reflect both the referral nature of the study center and the advanced disease profile of the population, may limit comparability with studies using more restrictive definitions. Although this variable was not associated with early complications in our analysis, its high prevalence may affect the external validity of the findings.

## 6. Conclusions

In this prospective cohort conducted in a Mexican hospital with limited resources, initiation of urgent-start peritoneal dialysis within 72 h after catheter placement was associated with a higher frequency of early mechanical complications, particularly pericatheter leakage. This association may reflect both the clinical severity of patients requiring earlier dialysis and technical factors related to catheter placement and dialysis prescription.

Whenever clinically feasible, delaying dialysis initiation beyond 72 h after catheter insertion and optimizing catheter placement techniques and dialysis prescriptions may help reduce early complications. Further multicenter studies with larger cohorts and longer follow-up are needed to validate these findings and guide the development of standardized protocols for urgent-start peritoneal dialysis programs.

## Figures and Tables

**Table 1:** Baseline characteristics of the population.

Variable	Results N = 65
Sex (female)	33 (50.8%)
Diabetes	63 (96.9%)
Hypertension	63 (96.9%)
Previous Abdominal Surgery	64 (98.5%)
Ischemic heart disease	8 (12.3%)
Obesity	18 (27.7%)
Etiology of CKD	
Diabetes	55 (84.6%)
Autoimmune disease	3 (4.6%)
Obstructive uropathy	4 (6.2%)
Others	3 (4.6%)
Reason for urgent initiation	
Uremic syndrome	42 (64.6%)
Refractory edema	19 (29.2%)
Hyperkalemia	4 (6.2%)
Age (years)	54.75 ± 9.62
Weight (kilograms)	72.48 ± 12.20
Height (centimeters)	160 ± 11.20
CKD time (months)	10 (1.5–36)
Dialysis start time (hours)	37 (31.5–44.5)
Average volume for exchange (mL)	1000 (1000–1500)
Number of exchanges per day	6 (5–8)
Uresis (mL)	725 (600–925)
Creatinine (mg/dL)	10.38 ± 4.34
BUN (mg/dL)	115 ± 47
Sodium (mmol/L)	133 ± 7.5
Potassium (mmol/L)	5.3 ± 1.28
Chloride (mEq/L)	100 ± 7.62
Phosphorus (mg/dL)	8.97 ± 3.11
Calcium (mg/dL)	7.47 ± 1.30
Magnesium mg/dL	2.32 ± 0.5
Glucose (mg/dL)	125 ± 51
Hemoglobin (grams/liter)	8.74 ± 1.70
Platelets 10^3^/µL	279 ± 114
Leukocytes 10^3^/µL	9.82 ± 4.16
BMI (kg/m^2^)	28.24 ± 4.47
Outcomes	29 (44.6%)
Type of complications	
Leak	12 (18.5%)
Catheter translocation	4 (6.2%)
Drainage failure	7 (10.8%)
Peritonitis	3 (4.6%)
Tunnelitis	3 (4.6%)

CKD: chronic kidney disease, BUN: blood urea nitrogen, BMI: body mass index, mg/dL: milligrams/deciliters, kg/m^2^: kilograms per meter^2^, mL: milliliters.

**Table 2:** Comparison by complication or no complication.

Variable	Complication N = 29	Without Complication N = 36	*p* Value
Female	16 (55.2%)	17 (47.2%)	0.62
Previous abdominal surgery	29 (100%)	35 (97.2%)	0.554
Reason for urgent initiation			
Uremic syndrome	20 (69%)	22 (61.1%)	0.717
Refractory edema	7 (24.1%)	12 (33.3%)	
Hyperkalemia	2 (6.9%)	2 (5.6%)	
Time			
<48 h	23 (79.3%)	18 (50%)	0.02
≥48 h	6 (20.7%)	18 (50%)	
<72 h	27 (93.1%)	26 (72.2%)	0.03
≥72 h	2 (6.9%)	10 (27.8%)	
Obesity	10 (34.5%)	9 (25%)	0.426
Death	3 (10.3%)	3 (8.3%)	0.603
Age (years)	55.31 ± 9.66	50.56 ± 1.94	0.6
Weight (kg)	72.47 ± 12.24	69.50 ± 17.44	0.425
Height (cm)	159.55 ± 11.04	160.53 ± 9.58	0.709
CKD time (months)	8 (1–36)	15 (11.5–42)	0.58
Dialysis start time (h)	37 (33–43)	49 (36–78)	0.014
Average volume for exchange (mL)	1000 (1000–1000)	1000 (1000–1000)	0.21
Number of exchanges per day	6 (5–8)	6 (6–6)	0.989
Uresis (mL)	700 (600–900)	725 (550–1000)	0.785
Creatinine (mg/dL)	9.77 ± 4	9.77 ± 5.12	0.998
BUN (mg/dL)	109.07 ± 50.86	99.14 ± 39.8	0.394
Glucose (mg/dL)	126.7 ± 50.85	130.21 ± 63.93	0.806
BMI (kg/m^2^)	28.64 ± 5.37	26.81 ± 5.73	0.189
Sodium (mmol/L)	133.85 ± 7.4	133.44 ± 7.76	0.83
Potassium (mmol/L)	5.1 ± 1.22	5.02 ± 1.42	0.611

CKD: chronic kidney disease, BUN: blood urea nitrogen, BMI: body mass index, mg/dL: milligrams/deciliters, kg/m^2^: kilograms per meter^2^, mL: milliliters.

## Data Availability

The datasets generated during and/or analyzed during the current study are available from the corresponding author upon reasonable request.

## Supplementary Materials

The following supporting information can be downloaded at: https://www.mdpi.com/article/10.3390/clinpract16040073/s1, Supplementary Material S1 (The STROBE reporting checklist); Supplementary Figure S1 (Study flow diagram) and Supplementary Figure S2 (BUN levels according to timing of dialysis initiation).

## Abbreviations

The following abbreviations are used in this manuscript:

UPD	Urgent peritoneal dialysis
CKD	Chronic kidney disease
GFR	Glomerular Filtration Rate
INEGI	National Institute of Statistics, Geography and Informatics
UDI	Urgent dialysis initiation
IMSS	Mexican Institute of Social Security
ISPD	The International Society for Peritoneal Dialysis
KRT	Kidney replacement therapy
PD	Peritoneal dialysis
