# Supplementary material for: Complications Related to Urgent Initiation of Peritoneal Dialysis in a Mexican Hospital with Limited Resources: A Prospective Cohort
Source: Clin Pract. 2026 Apr 13;16(4):73. doi: 10.3390/clinpract16040073 (PMC13115390; doi:10.3390/clinpract16040073)
Supplement: Supplementary file 1 [file clinpract-16-00073-s001.zip › clinpract-4091929-supplementary.pdf]

Supplementary Material S1

The STROBE reporting checklist

For checking that observational epidemiology research articles can be understood and used by everyone

> Note
>
> If you have not used a reporting guideline before, read about how and why to use them and check whether STROBE is the most applicable reporting guideline for your work.
>
> Reporting guidelines are most useful when used early in research. When writing a manuscript or application, consider using the Full Guidance where you'll see explanations and examples for each item.
>
> After writing, demonstrate adherence by completing this checklist:
>
> 1. Specify where each item is described (see Note 1).
> 2. Cite this checklist (See Note 2).
> 3. Include your completed checklist as a supplement when submitting to a journal so that future readers can use it to find information.

	Item Description	Location (or reason for not reporting)
**Title and abstract**		
1a. Indicate the study's design	Indicate the study's design with a commonly used term in the title or the abstract.	Yes, it is a prospective cohort.
1b. Abstract	Provide in the abstract an informative and balanced summary of what was done and what was found.	Yes, paragraph 1
**Introduction**		
2. Background / rationale	Explain the scientific background and rationale for the investigation being reported.	Yes, paragraphs 2 and 3
3. Objectives	State specific objectives, including any prespecified hypotheses.	Yes, paragraph 5
**Methods**		
4. Study design	Present key elements of study design early in the paper.	Yes, paragraph 6
5. Setting	Describe the setting, locations, and relevant dates, including periods of recruitment, exposure, follow-up, and data collection.	Yes, paragraph 6
6a. Eligibility criteria	**Cohort study:** Give the eligibility criteria, and the sources and methods of selection of participants. Describe methods of follow-up. **Case-control study:** Give the eligibility criteria, and the sources and	Yes, paragraph 6

	methods of case ascertainment and control selection. Give the rationale for the choice of cases and controls. **Cross-sectional study:** Give the eligibility criteria, and the sources and methods of selection of participants.	
6b. Matching criteria	**Cohort study:** For matched studies, give matching criteria and number of exposed and unexposed. **Case-control study:** For matched studies, give matching criteria and the number of controls per case.	No, it is a an observational study.
7. Variables	Clearly define all outcomes, exposures, predictors, potential confounders, and effect modifiers. Give diagnostic criteria, if applicable.	Yes, paragraph 7
8. Data sources / measurement	For each variable of interest give sources of data and details of methods of assessment (measurement). Describe comparability of assessment methods if there is more than one group.	Yes, paragraph 7
9. Bias	Describe any efforts to address potential sources of bias.	No, To minimize potential biases, we applied the following measures:<br>• Standardized data collection forms were used to reduce observer variability.<br>• Only patients with complete 30-day follow-up were included in outcome analyses.<br>• Multivariate models were adjusted for clinically relevant confounders, including age, diabetes, hypertension, and baseline albumin
10. Study size	Explain how the study size was arrived at.	No, A sample size calculation was not performed a priori; the sample was determined by convenience, based on the total number of eligible patients within the study period. However, post hoc power analysis was conducted to assess the strength of observed associations.
11. Quantitative	Explain how quantitative variables were handled in	Yes, paragraph 7

variables	the analyses. If applicable, describe which groupings were chosen, and why.	
12a. Statistical methods	Describe all statistical methods, including those used to control for confounding.	Yes, paragraph 7
12b. Statistical methods – subgroups and interactions	Describe any methods used to examine subgroups and interactions.	Yes, we did a Stratification performed by complication groups
12c. Statistical methods – missing data	Explain how missing data were addressed.	No, because we had just missed data with albumin and we decide to eliminate the variable
12di. Statistical methods – loss to follow-up	**Cohort study:** If applicable, describe how loss to follow-up was addressed.	Yes, paragraph 6
12dii. Statistical methods – matching cases and controls	**Case-control study:** If applicable, explain how matching of cases and controls was addressed.	No, because it is a prospective cohort.
12diii. Statistical methods – sampling strategy	**Cross-sectional study:** If applicable, describe analytical methods taking account of sampling strategy.	No, because it is a prospective cohort.
12e. Statistical methods – sensitivity analyses	Describe any sensitivity analyses.	Yes, paragraphs 7 and 8
**Results**		
13a. Participant numbers	Report the numbers of individuals at each stage of the study—e.g., numbers potentially eligible, examined for eligibility, confirmed eligible, included in the study, completing follow-up, and analysed; Consider use of a flow diagram.	Yes, paragraphs 6 and 8
13b. Participants – non-participation	Give reasons for non-participation at each stage.	Yes, paragraph 6
13c. Participants – flow diagram	Consider use of a flow diagram.	Yes, we added as a supplementary material
14a. Descriptive data – participant characteristics	Give characteristics of study participants (e.g., demographic, clinical, social) and information on exposures and potential confounders. Present the information in a table.	Yes, paragraph 8
14b. Descriptive data – missing data	Indicate the number of participants with missing data for each variable of interest.	No, because we had just missed data with albumin and we decide to eliminate the variable
14c. Descriptive data – follow-up	**Cohort study:** Summarise follow-up time—e.g., average and total amount.	Yes, paragraphs 6 and 8

time		
15. Outcome data	**Cohort study:** Report numbers of outcome events or summary measures over time. **Case-control study:** Report numbers in each exposure category, or summary measures of exposure. **Cross-sectional study:** Report numbers of outcome events or summary measures.	Yes, table 2 and 3
16a. Main results	Give unadjusted estimates and, if applicable, confounder-adjusted estimates and their precision (e.g., 95% confidence intervals). Make clear which confounders were adjusted for and why they were included.	Yes, Table 2 and 3<br>Paragraphs 8, 9, 10, 11, and 12.
16b. Main results – category boundaries	Report category boundaries when continuous variables were categorised.	No, because we only categorized by discrete or qualitative variables.
16c. Main results – risk	If relevant, consider translating estimates of relative risk into absolute risk for a meaningful time period.	No
17. Other analyses	Report other analyses done—e.g., analyses of subgroups and interactions, and sensitivity analyses.	Yes, paragraphs 9 and 10.
**Discussion**		
18. Key results	Summarise key results with reference to study objectives.	Yes, paragraphs 13, 14, 15, 16, 17, 18, 19 and 20.
19. Limitations	Discuss limitations of the study, taking into account sources of potential bias or imprecision. Discuss both direction and magnitude of any potential bias.	Yes, paragraph 23
20. Interpretation	Give a cautious overall interpretation considering objectives, limitations, multiplicity of analyses, results from similar studies, and other relevant evidence.	Yes, paragraph 23
21. Generalisability	Discuss the generalisability (external validity) of the study results.	Yes, paragraph 23
**Other information**		
22. Funding	Give the source of funding and the role of the funders for the present study and, if applicable, for the original study on which the present article is based.	No, without financing.

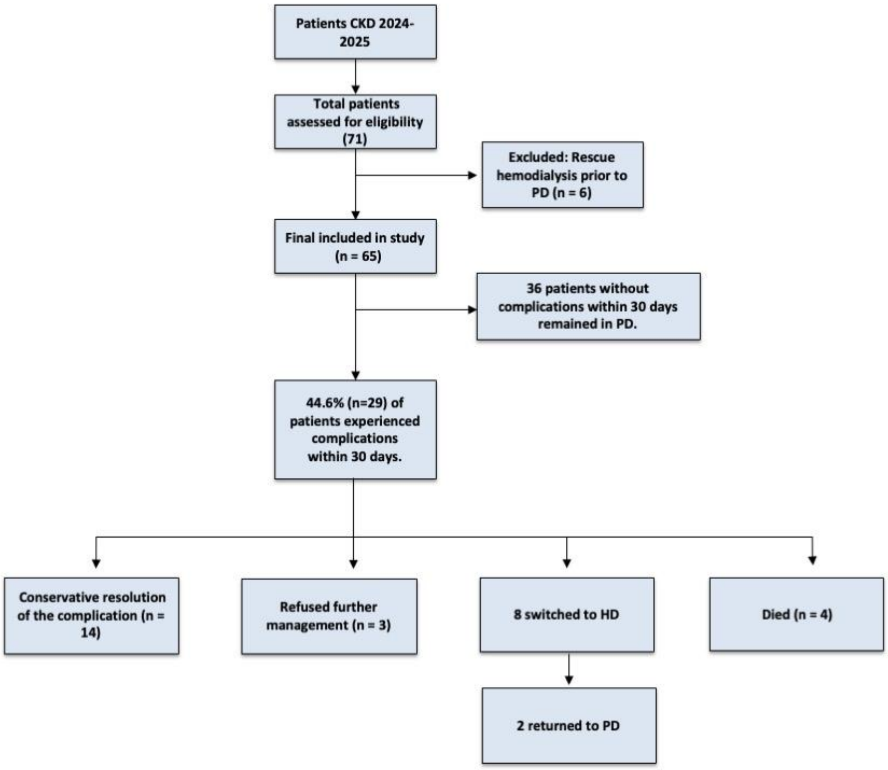

Supplementary Figure S1. Study flow diagram. Flow diagram of patient selection and clinical outcomes. A total of 71 patients with chronic kidney disease requiring urgent-start peritoneal dialysis (PD) were assessed for eligibility between 2024 and 2025. Six patients were excluded due to the need for rescue hemodialysis prior to PD initiation, resulting in a final cohort of 65 patients. Within 30 days, 29 patients (44.6%) developed complications, while 36 remained complication-free on PD. Among those with complications, 14 were managed conservatively, 3 declined further management, 8 required transfer to hemodialysis (HD), and 4 died. Of those transferred to HD, 2 subsequently returned to PD.

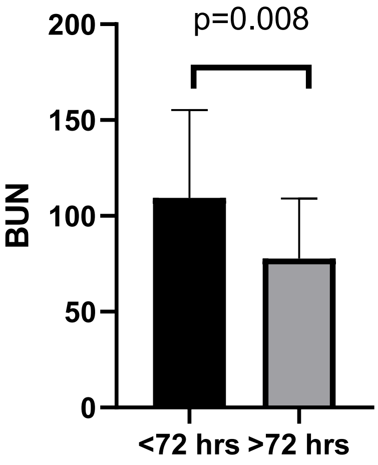

Supplementary Figure S2. Blood urea nitrogen levels according to timing of dialysis initiation. Comparison of baseline blood urea nitrogen (BUN) levels according to timing of peritoneal dialysis initiation (<72 hours vs ≥72 hours after catheter placement). Patients initiating dialysis within 72 hours exhibited significantly higher BUN levels, reflecting greater metabolic severity at presentation (mean ± SD: 109.44 ± 45.72 mg/dL vs 77.64 ± 31.39 mg/dL; $p = 0.008$). Statistical significance was assessed using an independent samples t-test.
